# A Novel Metal Transporter Mediating Manganese Export (MntX) Regulates the Mn to Fe Intracellular Ratio and *Neisseria meningitidis* Virulence

**DOI:** 10.1371/journal.ppat.1002261

**Published:** 2011-09-29

**Authors:** Frédéric J. Veyrier, Ivo G. Boneca, Mathieu F. Cellier, Muhamed-Kheir Taha

**Affiliations:** 1 Institut Pasteur, Infection Bactériennes Invasives, Dept. Infection et Epidémiologie, Paris, France; 2 Institut Pasteur, Groupe Biologie et génétique de la paroi bactérienne, Dept. Microbiologie, Paris, France; 3 INSERM, Groupe Avenir, Paris, France; 4 INRS-Institut Armand-Frappier, Laval, Quebec, Canada; Northwestern University Feinberg School of Medicine, United States of America

## Abstract

*Neisseria meningitidis* (Nm) and *N. gonorrhoeae* (Ng) are adapted to different environments within their human host. If the basis of this difference has not yet been fully understood, previous studies (including our own data) have reported that, unlike Ng, Nm tolerates high manganese concentrations. As transition metals are essential regulators of cell growth and host pathogen interactions, we aimed to address mechanisms of Nm Mn^2+^ tolerance and its pathogenic consequences. Using bioinformatics, gene deletion and heterologous expression we identified a conserved bacterial manganese resistance factor MntX (formerly YebN). The predicted structure suggests that MntX represents a new family of transporters exporting Mn. In the *Neisseria* genus, this exporter is present and functional in all Nm isolates but it is mutated in a majority of Ng strains and commonly absent in nonpathogenic species. In Nm, Mn^2+^ export via MntX regulates the intracellular Mn/Fe ratio and protects against manganese toxicity that is exacerbated in low iron conditions. MntX is also important for *N. meningitidis* to resist killing by human serum and for survival in mice blood during septicemia. The present work thus points to new clues about Mn homeostasis, its interplay with Fe metabolism and the influence on *N. meningitidis* physiology and pathogenicity.

## Author Summary

*Neisseria meningitidis* is an obligate resident of the human nasopharynx but can also be responsible for septicemia and meningitis. During our efforts to understand the specific selective pressure underwent by *N. meningitidis* to survive in its human niche, we have brought to light a new family of bacterial manganese-exporters (MntX) strongly conserved in *N. meningitidis* but often inactivated or absent in other *Neisseria* species. As iron, manganese is an essential metallo-nutrient for bacteria. Thus, the *N. meningitidis* need for a manganese-exporter seemed rather surprising. In fact, we were able to show that MntX is an important player in the regulation of the manganese/iron equilibrium and that this regulation via MntX is critical to survive in presence of manganese in particular when iron is rare. It is expected that excessive iron replacement by manganese into the active site of enzymes would handicap bacteria. Accordingly, MntX is required for full virulence of *N. meningitidis* in a mice model of septicemia or to resist killing by human serum. More generally, this equilibrium may be tightly regulated in other respiratory tract pathogens such as *S. pneumoniae* and therefore, interferences with this balance may be a promising strategy for infectious diseases therapy.

## Introduction

It is largely accepted that access to metals impacts on the equilibrium of host pathogen interface [1], [2]. In fact, bacteria must acquire nutrients for survival from the host environment during the course of the interaction. These nutrients comprise transition metals (such as Fe, Mn, Zn, Ni, Cu, Co and Mo) [3] which have the specific characteristic of an incompletely filled “d” orbital. This permits different state of oxidation (e.g. Fe^2+^ and Fe^3+^) and their use for proteins structural stabilization or as enzymes cofactors in a majority of metabolic processes [4]. Accordingly, transition metals are essential for the survival of bacteria. However, their accumulation can be toxic if the quantity, state of oxidation or intracellular localization and regulation are inadequate [5]. In these cases, metals can cause deleterious oxido-reduction reactions with proteins or other compounds (e.g. H_2_0_2_ and iron also known as Fenton reaction), generating toxic compounds (e.g. OH. OH^-^) that alter macromolecular structures such as proteins, membranes and DNA, leading to cell death [5]. In bacteria, this duality has forced selection of strategies to orchestrate essential transition metals homeostasis by sensing, acquiring, storing or, when needed, exporting them properly. On the host side, the duality of metal functionality is also true and metal homeostasis is also tightly controlled. Furthermore, prokaryote-eukaryote co-evolution has selected immune strategies aimed at controlling metals availability and restricting bacterial growth [3], [6], [7].

If the dominant role of iron in host-pathogen interactions has been extensively documented the impact of other transition metals and the interplay between their metabolisms are just emerging. One can cite the example of Mn for which the importance in bacterial physiology and pathogenesis just became apparent [8], [9], [10]. In addition to its role as cofactor in several bacteria, manganese has been suggested to quench reactive oxygen species (ROS) [11], [12] which could be endogenous (generated during bacterial metabolism) or exogenous (host immune defense mechanism). Bacterial manganese importers have been shown to influence host-pathogen interactions but depending on the infectious model and type of pathogen they were found to either contribute or not to virulence [13], [14], [15], [16]. Their definition in terms of general bacterial pathogenicity determinants is therefore unclear. Besides, the homeostasis of manganese has to be considered in a more complex situation when several metals are acting in concert. Examples of metals interplay have been described with manganese control of bacterial iron homeostasis [17] or more importantly, adequate intracellular Mn/Fe ratio critical to resist to certain stress [18], [19], [20].

The impact of metal availability on pathogenesis is particularly exemplified by *Neisseria* human pathogens. The *Neisseria* genus is composed of bacteria which are part of the normal human microbiome and live in harmless symbiosis with humans. Unfortunately, in some cases this relation may evolve to parasitism. This is particularly the case of two species namely *N. meningitidis* and *N. gonorrhoeae.* These two closely related bacteria are exclusively found in humans but in different ecological niches consequently causing distinct diseases. *N. meningitidis* is frequently isolated from the upper respiratory tract of asymptomatic carriers but can also be the causative agent of life threatening invasive infections such as septicemia and meningitis. *N. gonorrhoeae* is isolated from the genitourinary tract and is the causative agent of gonorrhoeae a sexually transmitted disease generally characterized by a localized inflammation with, in some circumstances, severe consequences. In both cases, the importance of Fe acquisition for *Neisseria* pathogenesis has been previously established. As a matter of fact, deletion of genes encoding metallo-transporters [21], [22], [23], [24], [25] leads to a decreased of virulence in model of infection. Regarding *N. meningitidis*, an additional approach has been used by providing a compatible iron source that enhances virulence in a mice model [26], [27]. In contrast, the role of Mn and its potential interplay with Fe metabolism remains unclear in this pathogen.

Some studies have demonstrated that *N. gonorrhoeae* is more sensitive to manganese than *N. meningitidis* [28], [29], [30]. The work in this article aims to extend this observation by: 1) identifying the genetic determinant responsible for Mn sensitivity and 2) describing the impact of its alteration on *N. meningitidis* pathogenesis. Our hypothesis was that Mn homeostasis may be different between *Neisseria* spp., which evolved to fill distinct host niches, and these differences might give clues about *N. meningitidis* virulence.

## Results

### Manganese tolerance is an attribute conserved in *N. meningitidis*

**Figure 1:**
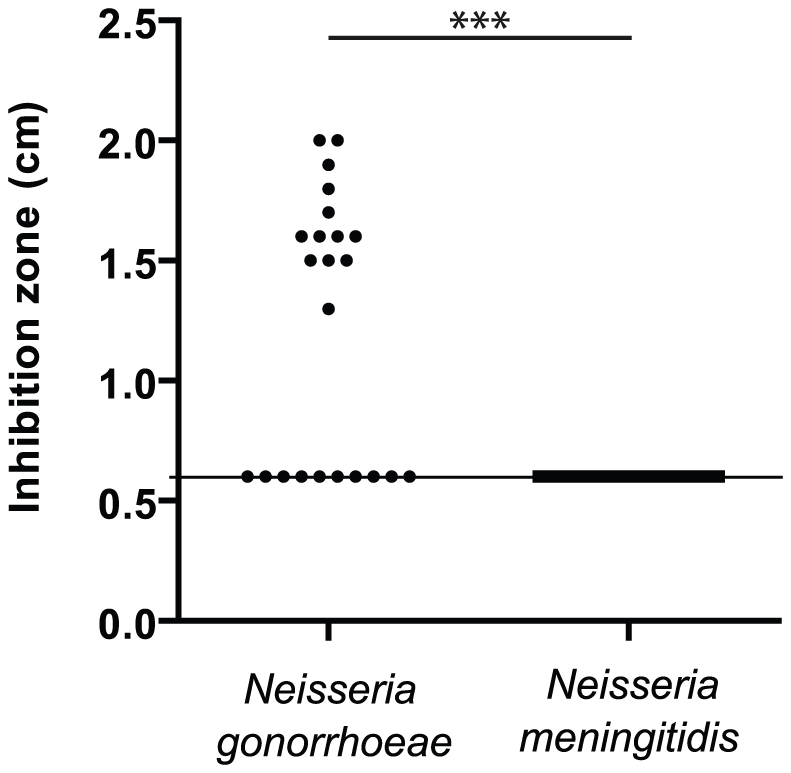
Resistance to manganese toxicity is a conserved trait in *Meningococcus.* Disk assay of the sensitivity of bacterial growth to 1M MnCl_2_ in GCB agar media was carried out for several clinical isolates of *N. gonorrhoeae* and *N. meningitidis*. (*** p<0.01)

To confirm that *N. gonorrhoeae* is more sensitive to manganese than *N. meningitidis* [28], [29], [30], we tested the sensitivity of a sample (≈20 strains) of each species isolated during the last twenty years by the Centre National de Reference des Meningocoques (Table S1). The Figure 1 presents Mn-dependent growth inhibition of these isolates measured by disk assay. Approximately 60% of strains of *N. gonorrhoeae* were Mn-sensitive compared to none in the case of *N. meningitidis* isolates. This result suggests that the capacity to resist to manganese toxicity is globally present in both species but that this capacity is strongly conserved only in *N. meningitidis*. In both bacteria, genes encoding the manganese/zinc transporter system MntABC are present and conserved [30], implying a similar capacity for manganese import. Thus we postulated the existence of a new actor in the manganese homeostasis.

### Search for a new Mn^2+^-regulated bacterial factors

Such an actor in manganese homeostasis should be regulated by Mn^2+^ to act when required. At the transcriptional level, the best known manganese regulators are the DtxR-related factor MntR [31] or Mur, derived from the general ferric uptake regulator (Fur) [17], [32]. Since, the genome of *N. meningitidis* does not code for these specialized metallo-regulators (our observations), we have preferred an alternative strategy which has consisted to first identify new actors in a model organism and to later verify their presence and evolution in the *Neisseria* genus. The bold speculation was that this factor may be present and regulated by MntR in other proteobacteria pathogens. We chose the phytopathogen *Xanthomonas campestris*, which possesses a simple predicted Mn regulon, including an *mntRH* locus, but lacks other Fur-derived transcription factor such as the oxido-metallo-regulator PerR or additional Mn importers such as the P-type ATPase MntA and the ABC-driven, periplasmic binding protein-based SitABCD transport system (our observation).

**Figure 2:**
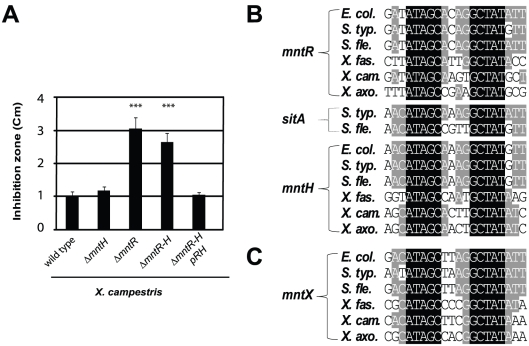
Search for Mn^2+^-regulated bacterial factor(s) in a simple model organism. A) *Xanthomonas campestris* MntR controls Mn transport: genetic studies of bacterial sensitivity to 1M MnCl_2_, measured by disk assay, revealed a novel Mn resistance factor, as *mntR* disruption increased Mn sensitivity independent of MntH import (*** p<0.01 compare to the wild type sensitivity). This experiment done in triplicates is representative of several experiments. B) Multiple sequence alignments of MntR binding sites found in the promoters of genes which contribute to Mn homeostasis (*mntH*, *mntR, sitA)* in γ-proteobacterial plant or animal pathogens (respectively *Escherichia coli*, *Salmonella typhimurium*, *Shigella flexneri* and *Xylella fastidiosa*, *X. campestris*, *X. axonopodis*). C) Sequence alignment of the MntR binding site detected upstream of one gene (*yebN* or XCC4075 herein referred to as *mntX*), using the PredictRegulon web site, and that is conserved γ-proteobacteria.

Thus, we deleted *mntH* (Mn-importer), *mntR* (Mn-regulator) or both genes and measured Mn sensitivity by disk diffusion assay. The results are illustrated in Figure 2A. The *mntH* deletion did not alter growth in the presence of Mn. In contrast, a drastic Mn-dependent growth inhibition was observed when *mntR* was deleted. First, we attributed this effect to an over-expression of MntH in absence of MntR but the double mutant Δ*mntH-R* was as affected as the single Δ*mntR* mutant. These results argued for the presence of at least one other MntR-regulated actor in manganese homeostasis in *X. campestris*.

To identify candidates, we used MntR-regulated promoters [33], [34], [35] to derive a matrix for MntR binding site (Figure 2B), which allowed consistent detection of one DNA motif (Figure 2C) in the 5′ region of a *Xanthomonas* gene termed XCC4075 (formerly *yebN*; in this article designated *mntX*).

### MntX is a conserved proteobacterial Mn-resistance factor not functional in a majority *N. gonorrhoeae* strains

#### In *X. campestris*

**Figure 3:**
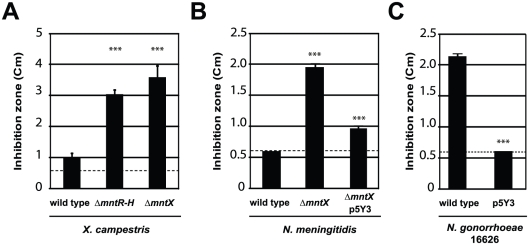
MntX which confers Mn resistance, is often not functional in *N. gonorrhoeae*. Disk assay of bacterial sensitivity to 1M MnCl_2_ was carried out for A) *X. campestris wild type* and mutants lacking *mntH and mntR or mntX.* B) *N. meningitidis* MC58 *wild type,* lacking *mntX* or back complemented (insertion of p5Y3 by double recombination). C) *N. gonorrhoeae* 16626 *wild type* or transformed with *mntX* from *N. meningitidis*. These experiments done in triplicates are representative of several experiments (*** p<0.01 compare to the wild type sensitivity).

We constructed a *mntX* deletion mutant and tested its manganese tolerance. The Figure 3A presents the results of this analysis and revealed that the *mntX* deletion leads to an increased manganese sensitivity as compared to the wild type bacteria. From this result, it can be concluded that *mntX* encodes a Mn-resistance factor in *X. campestris* (MntX*_Xc_*). In support of this interpretation, the comparison between Δ*mntX,* Δ*mntR*, Δ*mntR-mntX* and Δ*mntR-mntH-mntX* showed the dominant role of MntX*_Xc_* in conferring Mn sensitivity and its regulation by MntR (Figure S1).

#### In Neisseria

We next searched for possible homologs of MntX*_Xc_*, in the *Neisseria* genomes and whether genetic variation could explain the difference in manganese sensitivity between *N. meningitidis* and *N. gonorrhoeae*. A gene encoding a similar protein (48% overall identity, 64% similarity) was found in *N. meningitidis* (8 out of the 8 sequenced strains). Additionally, *mntX* was present in the genome of the closely related species *N. cinerea* (1/1), *N. polysaccharea* (1/1), *N. lactamica* (2/2) and *N. gonorrhoeae* (16/16). But we noticed in this latter organism that the gene was frameshift mutated in 66% of sequenced strains (once 7/16 or twice 4/16). Also, the gene was absent from the more distantly related species *N. elongata* (1/1), *N. flavescens* (2/2), *N. mucosa* (1/1), *N. sicca* (1/1), *N. subflava* (1/1) and *N. sp.* oral taxon 014 str. F0314 (1/1).

To test that *N. meningitidis mntX* (MntX*_Nm_*) encodes also a Mn resistance factor we constructed a deletion mutant, Δ*mntX*, which showed increased sensitivity toward manganese in disk diffusion assay (Figure 3B). Notably, inactivation of *mntX* did not increase sensitivity toward other metal salts such as FeSO_4_, NiCl_2_ and ZnCl_2_ for both *X. campestris* and *N. meningitidis* homologs (data not shown). Lastly, to determine whether *N. gonorrhoeae mntX* mutation (*mntX_Ng_*) was the cause of the difference in manganese sensitivity observed between both species (Figure 1), we replaced *mntX_Ng_* (frameshift mutated) by *mntX_Nm_* (via p5Y3::Km) in one of the sensitive strain of *N. gonorrhoeae* (16626). As shown in the Figure 3C, complementation by *mntX_Nm_* restored manganese tolerance in the *N. gonorrhoeae* sensitive strain 16626. Overall these results strongly suggest that a manganese resistance factor is conserved in distant species and that specific evolutionary events explain the difference in sensitivity of two closely related *Neisseria*.

### MntX represents a novel family of prokaryotic metal transporters

**Figure 4:**
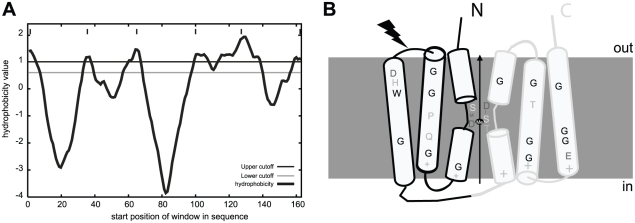
MntX identifies a family of predicted bacterial metal transporters. A) MntX*_Xc_* hydrophobicity plot based on the Goldman, Engelman, Steitz hydropathy scale, using core and wedge windows of 14 and 4 a.a. residues, respectively. B) Proposed model of structure-function relationships in MntX deduced from transmembrane topology prediction and evolutionary sequence analyses. MntX inverted structural symmetry and pattern of sequence conservation suggest a functional interface that may act as a conformational switch for metal transport. The black lightening form represents the localization of the truncation of MntX*_Ng_*.

To our knowledge MntX*_Nm_* and MntX*_Xc_* are the first members of a large family of highly hydrophobic putative membrane proteins (Figure 4A) of previously unknown function. As a first clue toward the mechanism of MntX-mediated manganese tolerance, we examined the phylogenetic distribution of 202 MntX homologues by Neighbor Joining (NJ) analysis [36]. The result, presented as a tree topology in the Figure S2A, shows significant discrepancy between the phylogenetic and taxonomic distributions of the sequences studied. The simplest explanation can be that *mntX* were frequently transmitted by horizontal gene transfer. Nevertheless, MntX*_Xc_* and MntX*_Nm_* are found in separate subgroups among most of the other proteobacterial sequences from α-, β- and γ-divisions.

To gain functional information on MntX, we generated logos of aligned protein sequences presented in Figure S2B. These graphical representations of amino acid frequencies by position allow simultaneous visualization of patterns and variations among MntX homologues that correspond to selected phylogenetic groups [37]. To identify features typical of the MntX family, we also included a phylogenetic outgroup of divergent sequences (in dark blue) [38]. Transmembrane segments (TMS) are the most conserved parts of the molecule and logos were obtained for each (Figure S2B, TMS1-3, top; TMS4-6, bottom). Examining these logos provided important structural information that is summarized in the predicted topology presented in Figure 4B.

First, similar patterns of conservation between TMS1-3 and TMS4-6 could be observed (underlined in the Figure S2B). This corresponds to the two directly repeated Domains of Unknown Function 204 (DUF204) [39] that form the MntX structure. In addition, conserved accumulations of positive charges found between TMS1/2, 3/4 and 5/6 (Figure S2B) were detected. Based on the ‘positive-inside’ rule, it was predicted that the two halves may adopt an inverted transmembrane configuration forming an overall pseudo-symmetric structure (Figure 4B). We also noticed periodicity of residue conservation in TSM2/3 and TSM5/6 compatible with α-helical structure and also the abundance of conserved glycine implying tight inter-helix packing interactions [40]. In comparison, the sequence conservation pattern in TMS1&4 is more local and central and involves adjacent residues which could form short extended segments. As reviewed in [41], such a configuration of repeated structures with a central extended segment is a characteristic of several membrane transporters. In these cases, the extended segments may provide contact points for substrate binding. In accordance with this hypothesis, MntX putative extended peptides (TMS1&4 as schematized in Figure 4B) contain invariant D residues which are know to be preferred ligands for divalent metals and polar moieties (S, T) also known to interact with inorganic cations [42]. In conclusion, this structural prediction suggests that MntX family is composed of metallo-transporters with conserved features putatively implicated in metal binding and transport

### MntX is an active manganese exporter

**Figure 5:**
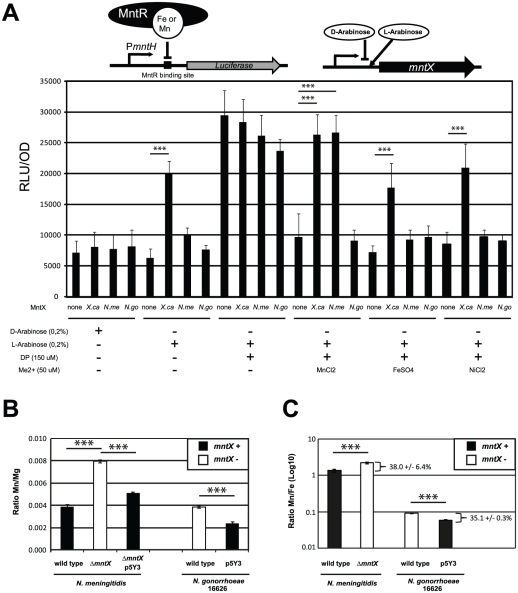
MntX is a manganese exporter. **A**) MntX depletes *E. coli* intracellular metal pools. The *E. coli* K-12 strain Δ*fur* P*mntH::luc* emits light upon intracellular metal depletion with the divalent metal chelator 2,2′-Dipyridyl (DP). This process is suppressed by co-incubation of DP with divalent metals, therefore allowing monitoring potential metal export activity. Plasmid-driven expression of MntX was induced with L-arabinose. Each bar represents the average of four independent measurements and is representative of several experiments **B** and **C**) ICP-MS quantification of divalent metals contents (Mg, Mn, and Fe), for strains of *N. meningitidis* and *N. gonorrhoeae* harboring *mntX_Nm_* (black bars) or not (white bars) and grown in rich medium. The data are expressed in ratio Mn/Mg in B and Mn/Fe in C. Each bar represents the average of three independent measurements for *N. meningitidis* and two for *N. gonorrhoeae* (*** p<0.01).

MntX can protect bacteria against manganese toxicity and sequences analyses suggest transport function. To test whether MntX encodes a metallo-exporter, we first used heterologous expression in *E. coli* using a model strain that reports on gene expression controlled by the intracellular pools of metals. In this strain (schematized in the top of Figure 5A), the *mntH* Orf was replaced by the firefly luciferase Orf. Furthermore, it is in a Δ*fur* background. Hence, luminescence is repressed exclusively by MntR (Figure 5A bar 1), with Mn^2+^ as co-repressor, and less efficiently with other metals as Fe^2+^ [43] (Bergevin and Cellier, unpublished). In this system, incubation of bacteria with a membrane permeant Me^2+^ chelator (2,2′-dipyridyl: DP) [16], [44] rapidly de-repressed significantly *PmntH-luc* expression (Figure 5A bar 9), and this could be prevented by co-incubation with some Me^2+^ (Figure 5A bar 13). With this assay, it is expected that a difference in luciferase expression may be observed if metals are pumped out of the cell via an exporter (i.e. the metal will not reverse the effect of DP).

To measure metal export in this *E. coli* strain, we expressed MntX*_Xc_*, MntX*_Nm_* and MntX*_Ng_* (strain 16626; frameshift mutated) via a L-arabinose inducible promoter present in the pBad plasmid (depicted in the top of Figure 5A). As a negative control we used bacteria transformed with an empty plasmid.

In rich medium, (LB broth) metals are in sufficient amount to control the expression of luciferase. The basal expression (D-arabinose) without production of MntX proteins was similar in all strains (bar 1 to 4). In contrast, when the production of MntX was induced (bar 5 to 8) the expression of luciferase was significantly increased only in the case of MntX*_Xc_* (bar 6). This suggests that MntX*_Xc_* is strongly active and able to deplete enough intracellular metals for MntR to be free from all possible metal cofactors (including Fe^2+^) in this rich medium. Other strains, in particular the one harboring *mntX_Nm_* did not differently express the luciferase when the production of MntX was induced (bar 3 compared to 7). This implies that MntX*_Nm_* is not active or it is not able to deplete, in rich medium, all possible MntR metallo-cofactors (including Fe^2+^).

In metallo-depleted medium (using DP bars 9 to 12), a maximum expression of the luciferase, independent of the presence of MntX, was observed. To test export of specific metal, bacteria were co-incubated with a Me^2+^ concentration allowing to significantly reverse DP intracellular action (Mn^2+^ Fe^2+^ or Ni^2+^). This reversion of the DP effect with metals could be observed for strains carrying the empty plasmid (bars 13, 17 and 21) or the plasmid encoding MntX*_Ng_* (bars 16, 20 and 24). In contrast, this reversion of the DP effect by Mn^2+^, Fe^2+^ and Ni^2+^ was not observed for the strain expressing MntX*_Xc_* (bars 14, 18 and 22). On the other hand, the luciferase activity was de-repressed in presence of MntX*_Nm_* only when DP was co-incubated with Mn^2+^ (bar 15) but not with Fe^2+^ (Bar 19) or Ni^2+^ (bar 23). As a note, similar results were observed for MntX*_Xc_* and MntX*_Nm_* using up to 300 µM of Mn^2+^ or Fe^2+^ (Figure S3). Overall, these results strongly suggest that MntX*_Xc_* exports a broad variety of metals whereas MntX*_Nm_* exports only Mn^2+^ when expressed in *E. coli.*

To confirm these results and examine MntX*_Nm_* endogenous function we performed ICP-MS analyses of metal content in *N. meningitidis* and *N. gonorrhoeae* harboring a functional MntX or not. We quantified intracellular atoms of manganese using bacteria grown in rich media with added Mn and using Mg or Fe content for normalizing the metal measurements (Figure 5B and 5C). For both strains, the lack of MntX activity (white) correlated with ∼two fold increase in Mn intracellular content compared to their parental or complemented strains (black). Similar results were obtained when Mn intracellular content was normalized with Fe (Figure 5C) suggesting that MntX*_Nm_* did not interfere with iron homeostasis and support *E. coli* data showing lack of MntX*_Nm_* specificity for Fe. Thus *N. meningitidis* MntX native activity supports the prediction that it is a membrane *t*ransporter that e*x*ports *Mn* and was therefore renamed MntX.

Of note, the intracellular amount of Mn measured in WT *N. gonorrhoeae* and *N. meningitidis* were close (respectively 3.0×10^5^ and 6.5×10^5^ Mn atoms by bacteria). However, about 10-fold more Fe was associated with WT *N. gonorrhoeae* compared to WT *N. meningitidis* (respectively 3.9×10^6^ and 4.9×10^5^ Fe atoms by bacteria), suggesting unsuspected differences in metal homeostasis between these close relatives, including an unusually high Mn/Fe ratio for *N. meningitidis*.

### MntX regulates the intracellular Mn/Fe ratio in *N. meningitidis*

**Figure 6:**
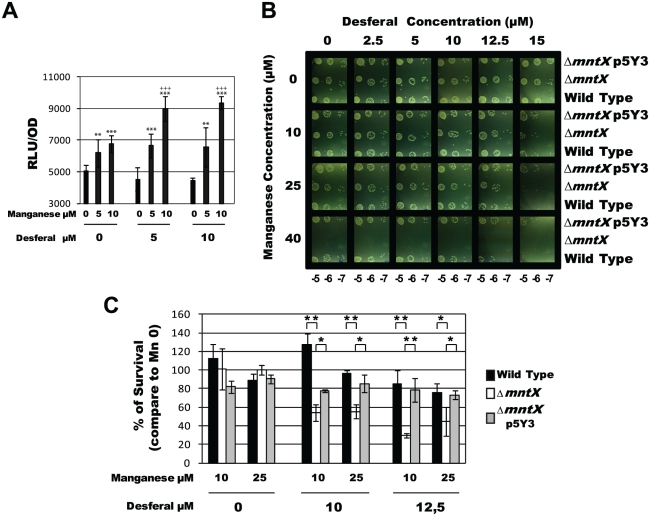
MntX protects *N. meningitidis* from Mn cytotoxicity exacerbated in low iron condition. **A**) Light emission normalized with OD, and done in triplicates, of *N. meningitidis* harboring P*mntX*::*luc* and grown in GCB agar for 4 hours containing indicated concentration of Desferal and manganese. (*** p<0.01, ** p<0.05 in comparison to the same Desferal concentration without Mn; +++ p<0.01 in comparison to the same Mn concentration without Desferal). **B**) Serial dilution plate assays were performed to compare the tolerance to iron chelation (Desferal) of *N. meningitidis* wild type strain, Δ*mntX* mutant and the complemented strain, in the presence of increased concentrations of MnCl_2_. **C**) Percentage of CFU growing on Desferal and manganese compared to those growing in the same concentration of Desferal but without manganese. Each bar represents the mean of three measurements. (*** p<0.01, ** p<0.05)

Manganese is typically considered as less toxic transition metal than iron (less Fenton's reaction) and beneficial in the context of an oxidative stress [45]. Thus, the existence of a manganese exporter in the genome of a bacterium which is only found in human, in metallo-restricted condition seemed rather surprising. Consequently, we tested if MntX could protect from oxidative stress. In *X. campestris* and *E. coli*, we were able to correlate the presence of MntX (expected to decrease intracellular manganese) with reduced resistance to organic peroxide toxicity (Figure S4). However, this was not true for *N. meningitidis* (Figure S4) as previously reported by others [30]. More importantly this trait was specific to *N. meningitidis* since *N. gonorrhoeae* was shown to accumulate manganese in defense against oxidative species [30], [46]. As *N. meningitidis* does not seems to rely on Mn to detoxify oxidative compounds, we reasoned that Mn over-accumulation may be deleterious, for example by competing with other essential metals when available in limited quantity (such as iron or zinc) for the binding to the active site of *N. meningitidis* proteins. Using a *N. meningitidis* strain harboring, in its native locus, the *mntX* promoter fused to the luciferase orf we observed that regulation of *mntX* was consistent with this hypothesis. The Figure 6A presents the key experiment which has consisted in growing *N. meningitidis* P*mntX*::luc in media with increased concentrations of manganese and Desferal (iron chelator). The results show that luciferase activity increased in presence of manganese but more importantly this expression was potentiated in low iron condition (i.e. manganese 10 µM and Desferal 5 µM) (Figure 6A).

To test a possible role for MntX under low iron/high manganese condition, we performed a serial dilution plate assay under increased concentrations of Desferal in combination with increased amount of manganese. The results presented in Figure 6B revealed that the growth of *N. meningitidis* harboring *mntX* (wild type or complemented) was neither affected by manganese alone (up to 40 µM) nor by Desferal alone (up to 15 µM). Most importantly, a drastic inhibitory effect of Desferal (15 µM) on the growth of *N. meningitidis* was strictly dependent on the presence of manganese (Figure 6, bottom right of panel B). This strongly suggests that with low iron availability manganese excess becomes lethal for *N. meningitidis*. Indeed, Δ*mntX* could not grow in manganese concentrations above 40μM, independently of iron concentration. To determine whether the Δ*mntX* mutation could increase sensitivity toward Desferal in the presence of sublethal Mn concentration (<40μM) we performed CFU counting experiments. The results presented in Figure 6C showed reduced survival in strains lacking MntX only in the presence of both Desferal and manganese but not in the presence of either compound alone.

Measurements of intracellular metal content showed that an active MntX decreases the Mn/Fe ratio by 38.0 ^+^/_-_ 6.4% in the case of *N. meningitidis* and 35.1 ^+^/_-_ 0.3% for *N. gonorrhoeae* (Figure 5C). All together, these data illustrate the importance of MntX to maintain an optimal Mn/Fe ratio in *N. meningitidis* via Mn export and in proportion with intracellular iron pool depletion. To test whether reduced fitness of bacteria lacking MntX under low iron/high manganese condition could be due to the abnormal replacement of Fe cofactors by Mn, we monitored the expression of Fur regulated genes. Accordingly, in absence of MntX, a miss-regulation of Fur regulated genes was observed in low iron/high manganese, illustrating these Mn interferences (Figure S5).

### MntX is important for virulence in sepsis models

**Figure 7:**
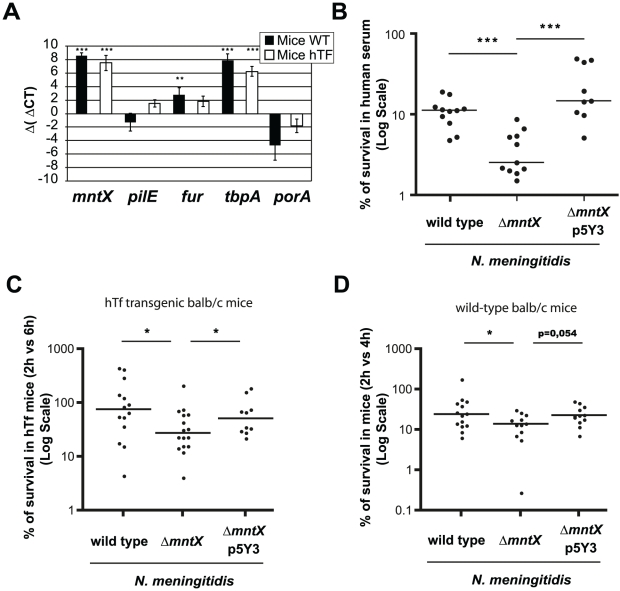
MntX is expressed during infection and is important to survive in serum. **A**) Expression of selected genes in bacteria from blood 6h post IP infection of hTf mice (black) or wild type mice (white), quantified by qRT-PCR. *gyrA* was used as endogenous house keeping gene whereas the reference is MC58 growing on GCB. Each bar represents the mean of three infections and representative of several experiments (*** p<0.001, ** p<0.005). **B**) Standard human serum bactericidal assays (50%) were performed for *N. meningitidis* wild type, Δ*mntX* or complemented. Each point represents a measure done in three independent experiments. (*** p<0.01) **C**) BALB/c mice expressing the human transferrin were infected intraperitoneally with 10^6^ bacteria from GCB agar. The graph represents the number of CFUs recovered from blood 6h post-infection compared to 2h p.i. and expressed in %. **D**) Similarly, BALB/c mice were infected intraperitoneally with 10^6^ bacteria from GCB agar containing 12.5 µM Desferal. The number of CFU recovered from blood 4h after infection was compared to 2h. For C and D, the graph presents the median % of CFU from two pooled independent experiments (* p<0.05).

Next we addressed the possibility that, since iron availability is restricted in the human body, *N. meningitidis* may require manganese export to survive in the host. First, we used the murine model of sepsis to determine if the gene was expressed in blood. To avoid residual expression due to culture media, we performed an *in vivo* passage (infecting mice with blood from a previously infected mouse) and extracted RNA. We employed two types of mice: wild type BALB/c mice and BALB/c mice expressing the human transferrin (hTf) [26]. *N. meningitidis* iron acquisition from transferrin is host-specific [47]. Therefore we used mice expressing the hTf (which is a compatible iron source) as an alternate model which sustains a better bacterial survival [26]. The Figure 7A presents the Δ (Δ Cycle threshold or Ct) of bacterial genes expressed in blood, normalized with *gyrA* and relative to the expression level upon growth on culture media. As a note, the Ct is defined as the number of cycles required for the fluorescent signal to cross the threshold. In this experiment, we measured the expression of *mntX* and genes encoding the pillin *pilE*, the global iron regulator *fur*, the transferrin binding protein A *tbpA* and the porin *porA*. The Δ (Δ Ct) of *mntX* was positive and similar between Balb/C and hTf mice. Thus, *mntX* was expressed in mouse blood suggesting that export of manganese is required in these conditions.

Secondly, we infected mice (BALB/c wild type or hTf) *i.p.* with *N. meningitidis* harboring or not *mntX* and determined the amount of bacteria in the blood at early (2h after infection) and later time point (4 h or 6h as indicated). The results of these experiments are expressed as % bacteria that survived in blood compared to those found at earlier time point (2 h) and are depicted in Figure 7C for hTf mice and Figure 7D for wild type mice. Median survival of *N. meningitidis mntX* mutant was significantly reduced compared to the wild type or complemented strains in both models of murine infection. Interestingly, the number of bacteria at 2h post-infection was similar independent of *mntX* indicating that the Δ*mntX* strain could still cross the epithelial barrier from the peritoneal cavity to the circulation. Nevertheless, to support a reduced survival of *N. meningitidis* Δ*mntX* in host blood, we assayed a human serum bactericidal activity with *N. meningitidis* wild type, Δ*mntX* or the complemented strain. As shown in Figure 7B, there was 3 to 4 times less *mntX* mutant surviving in the presence of this specific serum compared to the wild type or complemented bacteria, therefore supporting a role for manganese export in the survival of *N. meningitidis* during septicemia.

## Discussion

The present work establishes the manganese export function of MntX (formerly YebN), the first characterized members of a new family of metal exporter. Members of the MntX family have been found so far only in prokaryotes and phylogeny suggests pervasive *mntX* horizontal transfer. While these observations require functional validation, it is of interest that several known major pathogenic bacteria were found to harbor a homologue of MntX such as *Actinobacillus*, *Bacillus*, *Corynebacterium*, *Clostridum*, *Campylobacter*, *Fusobacteria, Legionella*, *Proteus*, *Pseudomonas* and enterobacteria (such as *Escherichia*, *Klebsiella*, *Yersinia, Salmonella*, *Shigella*) species (data not shown). In addition, members from this novel family display sequence characteristics that could be related to the evolution of their functional properties. MntX protein sequences are composed of direct repeats with inverted topology. A common evolutionary scenario reminiscent of the MFS, EmrE and LeuT superfamilies [48], [49], [50], [51] would propose that initially, an ancestral 3 TMS repeat (i.e., DUF204) could adopt inverted topologies and dimerize pseudo-symmetrically to catalyze a substrate translocation process. Subsequent steps of genetic fusion and evolution would have yielded a fixed 6TMS topology, and allowed substrate-driven divergence. MntX xenologs display appreciable sequence conservation despite inverted symmetrical organization; it is tempting to suggest that this family may have relatively recent origins, following the genetic fusion of two DUF204 domains. Future studies will establish whether *N. meningitidis* MntX Mn^2+^ selectivity can be related to site-specific sequence divergence within TMS and if other members could have evolved toward another specific metal export.

Beyond physico-chemistry, the novel family of manganese exporters MntX sheds new light on manganese impact on bacterial physiology. In this work, we studied the importance of Mn export via MntX in *N. meningitidis.* Unlike *N. gonorrhoeae, N. meningitidis* is not known to use Mn as quenching compounds during oxidative stress [30]. The selection of different strategies may reflect conditions encountered within their specific ecological niche. In fact, *N. meningitidis* (but also *N. lactamica*) are frequently isolated from the human nasopharynx, which is aerobic. The strong conservation of manganese export attribute in *N. meningitidis* suggests a damaging effect of manganese and a need for export in some conditions. It is generally recognized that the homeostasis of manganese and iron are linked; manganese importers expression is influenced by iron (e.g. via fur) [34] and, inversely, iron importer could be regulated by manganese [17]. In addition, several studies have recently correlated the ratio of Mn/Fe with the resistance toward specific stresses [18]. The results presented in this study, strongly suggest that in order to survive in iron restricted environment, *N. meningitidis* needs to regulate the Mn/Fe intracellular ratio and that MntX is contributing by exporting manganese. Consistent with this, inactivation of *mntX* reduces *N. meningitidis* virulence by limiting survival during septicemia in mouse model of infection. Similar attenuation has been observed using another human pathogen, *Streptococcus pneumoniae* and another family of manganese exporter. Lack of the CDF manganese exporter MntE reduced *S. pneumoniae* virulence by diminishing nasal colonization, blood invasion and mice mortality [10]. In the absence of MntX (or other manganese exporters), the replacement of iron by manganese in proteins may lead to suboptimal enzymatic activities or inadequate regulation. In stringent environments where bacteria need to be high-performers, a lack of competitiveness may reduce survival of the microorganism due to absence of vigorous responses.

However, a different strategy has been preferred for *N. gonorrhoeae* in order to survive in the genitourinary tract. This environment is anaerobiotic and rich in peroxides produced by host cells (e.g., neutrophils) and other host-adapted bacteria such as *Lactobacillus spp.* which compete for the same ecological niche [52]. Lactobacilli are well-known for their high intracellular manganese content and peroxide stress resistance [11]. It is therefore not surprising that *N. gonorrhoeae* uses also Mn-dependent peroxide detoxification strategies (reviewed in [53]). However, though Lactobacilli strategy relies on redundant Mn uptake systems [54], *N. gonorrhoeae* approach seems to exploit *mntX* gene mutation. Importantly, the *mntX* gene is not deleted from the genome and a reversion of the mutation(s) by phase variation may be possible if a modification of the local environment occurred (e.g., less H_2_0_2_). During our study we have also noticed that *N. gonorrhoeae* contains more intracellular iron than *N. meningitidis*. This result is similar to previous *N. gonorrhoeae* measurement [18] and may suggest that because *N. gonorrhoeae* accumulates more Fe than *N. meningitidis*, the Mn quantities tolerated in the cytoplasm are higher and the need for MntX is not as crucial as it is in *N. meningitidis*. In addition, this situation may have favored the use of a different strategy for peroxide stress protection aiming to increase manganese concentration during oxidative stress [46]. As our results reveal, this is not possible in *N. meningitidis* possibly due to lower intracellular iron content not permissive for this Mn-increase.

Bacterial homeostasis of iron and manganese or modulations of the Mn/Fe ratio are complex phenomena as they have to integrate information from both the extracellular environment and bacterial intrinsic physiology. In addition, the metabolism of other divalent metals such as Zn may also be coordinately regulated. In this sense, MntX and other manganese exporters have a crucial role to play in the interplay of metal metabolisms. In the future, it will be also important to precisely determine how the innate immune system is interfering with this ratio (versus iron alone) to understand how host defense could affect pathogenicity and if perturbation of this equilibrium could inspire new therapeutic strategies.

## Materials and Methods

### Ethics statement

This study was carried out in strict accordance with the European Union Directive 2010/63/EU (and its revision 86/609/EEC) on the protection of animals used for scientific purposes. Our laboratory has the administrative authorization for animal experimentation (Permit Number 75–1554) and the protocol was approved by the Institut Pasteur Review Board that is part of in the Regional Committee of Ethics of Animal Experiments of Paris region (Permit Number: 99–174). All surgery was performed under sodium pentobarbital anaesthesia, and all efforts were made to minimize suffering.

### Bacterial strains and culture conditions

*Xanthomonas campestris* pv *campestris* str. ATCC 33913 was purchased at the ATCC and was grown in SB (Silva Buddenhagen) medium at 30°C. *Neisseria meningitis* MC58 and clinical isolates of *N. meningitidis* and *N. gonorrhoeae* were obtained from the Centre National de Reference des Meningocoques (CNRM, Institut Pasteur, Paris). The description of these clinical isolates is provided in Table S1. All *Neisseria* were grown in GCB medium with Kellogg supplements. For cloning experiments, *E. coli* DH5α was grown at 37°C in Luria-Bertani Media (Difco). As required, antibiotics were added as follows: chloramphenicol (10 µg.ml^−1^), Spectinomycin (100 µg.ml^−1^), streptomycin (50 µg.ml^−1^), tetracyclin (10 µg.ml^−1^), gentamycin (30 µg.ml^−1^), kanamycin (50 µg.ml^−1^ for *E. coli*; 100 µg.ml^−1^ for *Neisseria* sp.) and Erythromycin (300 µg.ml^−1^ for *E. coli*; 3 µg.ml^−1^ for *Neisseria* sp.).

### Construction of *Xanthomonas campestris* mutants and complemented strain

The *mntH* (XCC2171) and *mntR* (XCC2170) genes are in the same locus and in an opposite direction in the genome of *X. campestris*. Therefore, to construct the plasmid used for double recombination, the 3′ part of *mntH* (amplified using the primers XcaSmaIF and XcampR2) was first inserted into pK18mobsacB [55] (gift of CVector) using the enzymes *Sma*I and *Eco*RI. Secondly, the plasmid generated, called pK18clon1, has served to generate all the other constructs by adding a specific 5′ part: as a part of *mntR* (to construct Δ*mntH-R*), or *mntR* and the rest of *mntH* (complemented) or the full *mntR* and a 5′ part of *mntH* (Δ*mntH*). These specifics 5′ parts were generated by specific enzymatic digestion of the same PCR product called PCR2 (obtained using XcaHindIIIF and XcaNoMutR primers). In more details, the construction of plasmids was as follow: 1) pK18*mntH*::Tet used to make the *mntH* deletion: PCR2 was inserted into pK18clon1 using *Sma*I and *Bam*HI. Following this, the tetracycline resistance cassette from p34S-tet [56] was inserted between the 5′ and 3′ fragments using *Sma*I. 2) pK18*mntH-R*::Gm used to make the *mntH-R* deletion: The chloramphenicol resistance cassette from p34s-Cm [56] was inserted into pK18clon1 using SmaI. The resulting plasmid was ligated with PCR2, after a digestion with *Sal*I and *Hind*III, to generate pK18*mntH-R*::Cm. Due to spontaneous resistance, the chloramphenicol resistance gene was changed by the gentamycin cassette. This one was extracted from p34S-Gm [56] using *Sma*I and inserted into pK18*mntH-R*::Cm digested with *Sma*I to generate the plasmid pK18*mntH-R*::Gm. 3) pRH used to complement the *mntR-H* deletion: PCR2 was inserted into pK18clon1 using *Pst*I and *Xba*I to generate pK18comp this has led to the reconstruction of the complete locus. The *mntH-R* genes were extracted to pK18comp using *Nhe*I and *Xba*I and were transferred into a *X. campestris* compatible plasmid, pBBR1-Tet digested by XbaI. Of note, pBBR1-Tet was generated by the ligation of pBBR1-Tp [57] digested with *Nhe*I and *Nco*I and treated with T4 DNA polymerase with the *Sma*I-digested tetracyclin cassette from p34S-Tet [56]. 4) pK18*mntR*::Sm used to make the *mntR* deletion: The plasmid pK18comp was digested with *Age*I and *Sal*I and, after treatment with T4 DNA polymerase, was ligated to a SmaI-digested spectinomycin resistance cassette from p34S-Sm3 [56]. 5) pK18*yebN*::Tet used to make the *yebN* deletion: A PCR product containing *yebN* and obtained with XCC4075F and XCC4075R was digested by *Nhe*I and *Xba*I and cloned in pK18mobsacB digested with the same enzyme. This new plasmid was digested by *Bgl*I (two sites in *yebN*), treated with T4 DNA polymerase and ligated with the tetracycline resistance cassette obtained by *Sma*I digestion of p34S-Tet, to finally generate pK18*yebN*::Tet [56]. All these plasmids were introduced in *X. campestris* using *E. coli* S17λpir as previously described [58]. The allelic exchange events were selected in SB medium containing 10% sucrose, ampicillin 100 µg.ml-1 (in order to inhibit *E. coli*) and the specific antibiotic for the deletion.

### Construction of *Neisseria* mutants and complemented strain

As a first step, the kanamycin and erythromycin resistance genes were amplified using Km6-KmUp and ERAM3-ERAMUp and subcloned into pGEM-T-easy (Promega) to give pGEM::Km and pGEM::Ery respectively. Secondly, the 5′ and 3′ DNA fragments were amplified from genomic DNA of the *N. meningitidis* MC58, using respectively KOYebN5′F-KOYebN5′R or KOYebN3′F-KOYebN3′R couple of primers. Lastly, the 5′ and 3′ DNA fragments were inserted sequentially into this pGEM::Km using respectively, *Nco*I and *Sph*I or *Nde*I and *Pst*I to finally generate p5′KOYebN3′::Km.

To construct the plasmid for the complementation, the full length *yebN* gene was amplified using YebNNsiIF and YebNPstIR and was inserted into p5′KOYebN3′::Km after a digestion with *Nsi*I and *Pst*I to generate p5Y3::Km. Subsequently, the kanamycine resistance gene has been removed and replaced by the erythromycin gene from pGEM::Ery using *Eco*RI to generate p5Y3::Ery. The luciferase was amplified using LucF and LucR, digested with *Nsi*I-*Pvu*II and ligated to p5Y3::Km digested with *Nsi*I-*Bsa*BI to generate p5L3::Km (luciferase under the control of the *yebN* promoter).

The p5′KOYebN3′::Km and p5L3::Km were then used to transform *N. meningitidis* MC58, and transformants were selected on GCB medium in the presence of 100 µg/ml Km. After verification of the deletion of *yebN*, the plasmid p5Y3::Ery was used to transform *N. meningitidis* MC58 Δ*yebN* and transformants were selected on GCB medium in the presence of 3 µg/ml Ery. Therefore, the functional *yebN* gene was introduced back in its original locus in the genome of *N. meningitidis* Δ*yebN*. Additionally, the plasmid p5Y3::Km was used to transform and complement *N. gonorrhoeae* 16626.

### Sensitivity to metal

First, to evaluate the metal sensitivity of *X. campestris*, *Neisseria sp.* and respective mutants, a disk assay of metal sensitivity was used. *X. campestris* and mutants were grown during a 16-h period at 30°C and 250 rpm. The cultures were then diluted in GTA broth to obtain a final OD_580_ of 0.1 and were further incubated at 30°C until OD_580_ 0.4. A volume of 1 ml was then taken, mixed with 9 ml of Top-SB medium and poured in 15 cm Petri plates containing 40 ml of solidified SB medium. After 15 min, a disk containing 10 µl of solution was placed on the center of the plates. For *Neisseria*, an aliquot of a 16h old culture (on plate) was taken and diluted on GCB media plus supplements to an OD_600_ of 1. Following this, 100 µl of this suspension were spread on a Petri dish containing 20 ml of GCB agar medium plus supplements. After 15 min, a disk containing 10 µl of solutions was deposed on the center of the plates. A standard t-test was done in order to assess statistically significant differences.

Secondly, growth sensitivity to the ratio manganese/iron was evaluated for the different strains of *N. meningitidis*. Serial dilutions of bacteria were spotted on plate containing increasing amount of Desferal and manganese. These plates were incubated overnight at 37°C after what, pictures were taken. To measure more precisely the effect of the ratio on bacterial growth, ≈250 bacteria were plated on GCB agar plate containing the indicated amount of manganese and Desferal, and the percentage of growth was calculated compared to the GCB agar with no manganese. A standard t-test was done in order to assess statistically significant differences.

### Measurement of intracellular metals depletion using *E. coli* Δ*fur*; p*mntH*-Luc over-expressing YebN

#### Construction of the plasmids

the *yebN* ORF from *X. campestris* was amplified using YNcoIF and YXbaIR whereas the ORF from *N. meningitidis* MC58 and *N. gonorrhoeae* 16691 was amplified using YebNF and YebNR. All these ORFs were cloned into pBAD24 using *Nco*I and *Xba*I.

#### Luciferase assay

Overnight cultures of each strain were diluted 1/40 into 1.8 ml of LB media and incubated during 2 h at 37°C with agitation. Following this, 200 µl of 10X cocktail (arabinose L or D; DP; Metals) was added at concentration indicated in Figure 5A. In parallel to the OD_600_ measurements, the bacterial lysis was done according to manufacturer recommendation (Luciferase assay system; promega) and directly after the addition of substrate, RLU were measured using LB96V MicroLumat Plus (Berthold) during 20 seconds and a standard t-test was applied to assess statistical significance.

### *yebN* gene expression *in vitro* and *in vivo*

For *in vitro* expression, we used the firefly luciferase fused to the *yebN* promoter and inserted in the genome as described above. The strain was grown on GCB agar plates overnight at 37°C and subculture in GCB agar plates containing the indicated concentration of MnCl_2_ and Desferal during 5 h. The bacterial layer was harvested in physiologic water to obtain an OD_600_. The luciferase activity was measured as described in the “Luciferase assay system” protocol (Promega) and a standard t-test was applied to assess statistical significance.

For *in vivo* expression, we performed qRT-PCR. Briefly, RNA was extracted using bacterial RNA protect and RNeasy Mini Kit (Qiagen) from blood of mice infected i.p. with 500 ml of blood from a mice previously infected i.p. with 10° bacteria. A passage from one mouse to another was done in order to attenuate the GCB media interference. qRT-PCR was performed using Power SYBR Green PCR master mix and StrepOne plus (Applied Biosystems) using the primers listed in table 1. In addition, a standard t-test was applied to assess statistically significant difference in comparison to the *gyrA* ΔCt.

**Table 1:** Oligonucleotide primers used in this study.

Primers name	Sequence (5′→ 3′)
XcampF	GC**CCATGG**CCAGCGAGATC
XcampR	CCC**TCTAGA**CTCTCACCCCA
XcampR2	G CT**TCTAGA**CCAACGCGAATTCCC
XcaHindIIIF	CGC**AAGCTT**GTCTGCCATATCAC
XcaNoMutR	ACACCAACACCACGTCCAGCG
XcaSmaIF	TTA**CCCGGG**CGGCATCAACC TG
XCC4075F	GCT**TCTAG**CACTGCGTGACGCGG
XCC4075R	CGC**GCTAGC**AATTTTCCACTGATCTC
YNcoIF	TGT**CCATGG**CTCCGCTCTCTATCG
*YXbaIR*	CTG**TCTAGA**CCGCGCCGCTGAGG
RTyebNF	TAGGCGGTTTTTATGCCAAG
RTyebNR	ATAGGCTTTCCCGTTTGCTT
furF	TGTACCGCATTTTGTTGGAA
furR	GTCGCCTTTGTCCAACTCAT
TbpAF	CTGAAATTGGGCGATAAGGA
TbpAR	CTGTTCCGCCATTTGTACCT
gyrAF	ACTCCGCTGCAAGACAGTTT
gyrAR	GAAAAGCGTACGTCGGGTAA
PorA101F	CGATAAACGAGCCGAAATC
PorAR	GCCGGCGTGGAAGGCAGGAA
KOYebN5′F	ATG**CTGCAG**GCGCGTTTGTTCAATTA
KOYebN5′R	ATA**CATATGCAT**ACCGCACCCGTCCT
KOYebN3′F	ATT**CCATGG**TGTCGGAAAATATAGTGGA
KOYebN3′R	ACA**GCATGC**GCAAAAAAGTCGCCGTC
YebNF	GCG**CCATGG**GTTTTTATGCTTTGCTC
YebNR	GAC**TCTAGA**GAATCAAACCCAAATGCG
YebNNsiIF	GGT**ATGCAT**TTTTATGCTTTGCTCTTG
YebNPstIR	AAAG**CTGCAG**TGATTCCGGATAAATT
LucNsiIF	ACC**ATGCAT**GACGCCAAAAACATAAAG
LucPstIR	ATA**CAGC** **TGCAG**CCTACAATTTGGACTTTC

### Determination of metal content using ICP-MS

The amount of metals accumulated in *Neisseria sp.* cells was determined by growing cells overnight on complete GCB and sub-culturing them in GCB agar plate containing 10 µM of Mn. After an incubation of 6h for *N. meningitidis* and 16h for *N. gonorrhoeae* (slow grower) the cells were resuspended in PBS and centrifuged. The pellets were subjected to acidic digestion using 500 µL of 65% acid nitric during one hour at 80°C. The preparation has been diluted with H_2_O (HPLC grade, Fisher) to obtain a concentration of 2% acid nitric. The samples were sent to Intertek Analytical Services (Chalon sur Saone; France) and analyzed by inductively coupled plasma mass spectrometry (ICP-MS) with Agilent 7500 cx (Agilent Technologies, USA). Results were expressed as the calculated ratio of number of atoms of the specified metal (MW used for Mn: 54,938) normalized with the number of Mg atoms (MW: 24,305) or Fe (MW: 55,845). Each strain has been cultured in triplicates for *N. meningitidis* and in duplicates for *N. gonorrhoeae* and a standard t-test was applied to assess significance.

### Sensitivity to human serum

To measure the percentage of survival, a specific human serum was selected due to its capacity to induce death in 30 to 50% of bacteria without addition of external source of complement. The assay was done as previously described [59] by incubating ≈1000 bacteria in HSSB^2+^ containing 50% of human serum during 30 min at 37°C. The results of several experiments (at least three) were pooled and one tailed Mann–Whitney test was used to determine statistical significance of observed differences (GraphPad Prism v5.0; GraphPad Software, CA).

### Survival in a mouse model of septicemia

The day of infection, inocula of *N. meningitidis* strains were prepared in PBS to obtain a final concentration of 2×10^7^ CFU/ml (OD600 0,1). Six week-old BALB/c (Janvier; France) or six to nine week-old human transferrin (hTf) transgenic mice [26] were infected by intraperitoneal challenge with 500 µl of inocula (1×10^7^ CFU). Bacterial counts in the blood were determined at 2h and 4h or 6h (for wild type or hTf mice respectively) after meningococcal challenge by plating serial dilutions of blood samples on GCB medium. Results are expressed in percentage of bacteria counted at 4 h or 6 h (for wild type or hTf mice respectively) compare to those counted at 2 h post infection. The results of several experiments (at least two) were pooled and one tailed Mann–Whitney test was used to determine statistical significance of observed differences (GraphPad Prism v5.0; GraphPad Software, CA).

### Bioinformatic analyses

To search for MntR regulated genes in bacterial genomes, we first sought the presence of putative conserved MntR binding sites using several predictive approaches. For this purpose, the sequence of the promoters of *mntH*, *mntR*, *sitA* from different proteobacteria were aligned using the AlignX program from VectorNTI (Invitrogen), and the resulting multiple alignment of similar motifs was used as a matrix to detect conserved binding sites in different genomes using the programs PredictRegulon [60] and PREDetector [61].

The protein hydropathy profile was calculated using the server TOPPRED [62] using a 20-residue long sliding window (core window: 14 residues, wedge windows: 4 residues). The default parameters suggested for predicting the presence of transmembrane segments in prokaryotic proteins were used, such as the Goldman, Engelman, Steitz hydropathy scale cut-off values (Lower, 0.6; putative; Upper, 1.0; certain). Protein transmembrane topology prediction was calculated using the MEMSAT-SVM server [63].

Molecular evolutionary sequence analyses were performed as previously described [38]. Sequences were classified by similarity clustering using CLANS [64] and multiple sequence alignment and phylogenetic analyses were conducted using the package Mega [36]. Briefly, BlastP searches using MntX revealed numerous similar bacterial sequences showing unexpected taxonomic distribution. To perform tractable sequence analyses we selected from databases of reduced complexity (entries <70% identity) candidate homologs co-linear with and displaying more than 30% identity to MntX. Clustering and tree-making approaches validated our sequence set as representative of the putative MntX family and including a possible phylogenetic outgroup [38]. To visualize MntX sequence conservation patterns we used a multiple-logo alignment tool [37] to compare the variability/homogeneity of aligned sequences representing hierarchically defined subclusters, such as the outgroup and the MntX family, as well as MntX family subgroups.

## Supporting Information

Figure S1 Disk assay of bacterial sensitivity to 1M MnCl_2_ for *X. campestris* wild type and mutants lacking all combination of *mntH*, *mntR* or *mntX*.
(EPS)

Figure S2 Phylogenetic patterns of MntX sequence conservation. **A)** MntX family tree topology established by the Neighbor-Joining method using unequal rate of evolution across sites and tested with 3000 bootstrap resampling. Tree branch colors reflect the taxonomic distribution of the sequences sampled: proteobacteria from α-, β-, γ-, δ- and ε-divisions; Gram positive bacteria, including Firmicutes and Actinobacteria; Green Non Sulfur bacteria, Cyanobacteria, Fusobacteria Chlorobi/Flavobacteriales/Bacteroidetes; Bacteria (Deferribacteres, Synergistetes, Lentisphaerae and Elusimicrobia) and Archaea (Euryarchaeota, Korarchaeota). The hierarchically ordered groups of sequences that were used for producing logos (shown in panel 2B): Pink (195 sequences: The MntH familly); blue (6 sequences: The outgroup); orange (32 sequences, including *Neisseria meningitis* and *Xanthomonas campestris* homologs indicated in green and purple, respectively). In addition each branch of the tree was colored in function of the phylogenic taxa. **B)** Sequence logos demonstrating preferential sequence conservation of MntX predicted TMS. MntX amino- and carboxy-internal repeats are shown superposed (top and bottom, respectively). MntX most conserved sites are boxed in red. Blue + indicate conserved positive charge clusters (predicted topogenic signals). In and Out symbols correspond to the positions of extra-membranous loops, respectively predicted to lie inside or outside the cell, and which are short and little conserved. Direct repetition (underlines in black) of a structural unit comprising 3TMS results in topologies that are inverted for MntX N- and C-halves. **C)** Proposed model of structure-function relationship in MntX transporter deduced from evolutionary sequence analyses and phenotypic observations. The black lightening form represents the localization of the truncation of MntX_Ng_.
(EPS)

Figure S3 Analyses of P*mntH*-luc activity in the presence of increasing amount of Mn **A)** or Fe **B)** indicated no impact of *N. meningitidis* MntX on the regulation mediated by Fe. Results are expressed in % of the maximal induction observed with DP alone.
(EPS)

Figure S4 Disk assay of bacterial sensitivity to tBOOH (organic peroxyde) for: **A)** *E. coli* EMG2 harboring the empty plasmid, pBAD encoding MntX_Xc_ or pBAD encoding MntX_Nm_. In this case, this assay as been done in presence of L-arabinose (0.2%). **B)** *X. campestris* wild type and mutant lacking *mntX.* **C)** *N. meningitidis* wild type, mutant lacking *mntX* and complemented strain. The concentration of tBOOH used has been 0.3M except for *X. campestris* which has been 0.5 M.
(EPS)

Figure S5 Missregulation of Fur-regulated genes in low Fe/high Mn conditions for *N. meningitidis* lacking MntX. Expression quantified by qRT-PCR, of selected genes for *N. meningitidis* wild type (black) or Δ*mntX* (white) growth during 6h in GCB with 12.5 µM Desferal and with 25 µM MnCl2. *gyrA* was used as the endogenous house keeping gene whereas the reference is MC58 growing on GCB with 12.5 µM Desferal only. Each bar represents the mean of three replicates. One can observe that the bacteria lacking MntX expressed significantly less *fur* and *tbpA* (Fur-regulated) in presence of manganese compare to the wild type strain. The same is not true for other genes not Fur regulated. (*** p<0.01).
(EPS)

Table S1 Clinical isolates description.
(XLS)
